# Using the weighted area under the net benefit curve for decision curve analysis

**DOI:** 10.1186/s12911-016-0336-x

**Published:** 2016-07-18

**Authors:** Rajesh Talluri, Sanjay Shete

**Affiliations:** Department of Biostatistics, The University of Texas MD Anderson Cancer Center, 1400 Pressler Dr, FCT4.6002, Houston, TX 77030 USA; Department of Epidemiology, The University of Texas MD Anderson Cancer Center, Houston, TX USA

**Keywords:** Decision curve analysis, Clinical decision making, Area under the curve, Net benefit curves, Threshold probabilities

## Abstract

**Background:**

Risk prediction models have been proposed for various diseases and are being improved as new predictors are identified. A major challenge is to determine whether the newly discovered predictors improve risk prediction. Decision curve analysis has been proposed as an alternative to the area under the curve and net reclassification index to evaluate the performance of prediction models in clinical scenarios. The decision curve computed using the net benefit can evaluate the predictive performance of risk models at a given or range of threshold probabilities. However, when the decision curves for 2 competing models cross in the range of interest, it is difficult to identify the best model as there is no readily available summary measure for evaluating the predictive performance. The key deterrent for using simple measures such as the area under the net benefit curve is the assumption that the threshold probabilities are uniformly distributed among patients.

**Methods:**

We propose a novel measure for performing decision curve analysis. The approach estimates the distribution of threshold probabilities without the need of additional data. Using the estimated distribution of threshold probabilities, the weighted area under the net benefit curve serves as the summary measure to compare risk prediction models in a range of interest.

**Results:**

We compared 3 different approaches, the standard method, the area under the net benefit curve, and the weighted area under the net benefit curve. Type 1 error and power comparisons demonstrate that the weighted area under the net benefit curve has higher power compared to the other methods. Several simulation studies are presented to demonstrate the improvement in model comparison using the weighted area under the net benefit curve compared to the standard method.

**Conclusions:**

The proposed measure improves decision curve analysis by using the weighted area under the curve and thereby improves the power of the decision curve analysis to compare risk prediction models in a clinical scenario.

**Electronic supplementary material:**

The online version of this article (doi:10.1186/s12911-016-0336-x) contains supplementary material, which is available to authorized users.

## Background

Risk prediction models are used to predict the probability of occurrence of future events for individuals based on several predictors. Predicting the risk of malignant events is of major importance for public health as such information can be used to improve outcomes and personalize clinical care. Risk prediction models have been developed for several cancers [1–3], a variety of conditions and general public health issues (e.g., hypertension, diabetes, cardiovascular disease, smoking experimentation) [4–7]. These risk prediction models are being constantly improved with the identification of new predictors (e.g., genetic markers) associated with the disease or condition of interest. However, assessing the contribution of these predictors in improving risk prediction is challenging.

The area under the receiver operating characteristic curve (AUC) is generally used to determine the predictive accuracy of a model [8]. The AUC provides a natural tool to select optimal models across all thresholds of sensitivity and specificity. However, in clinical settings, there may be situations in which a model with higher AUC may not be desirable. For example, if a treatment involves high risk, the model with a low false positive rate would be the best model to use despite its lower AUC compared to those for other models. Also, in clinical settings, the models need not be accurate at the extreme ranges. For example, consider a scenario in which, if the predicted probability for the disease were below 0.2, the individual would not be screened/treated, and if the predicted probability was above 0.8, the individual would be screened/treated. Small differences in the predicted probabilities from 2 competing models would not make a significant clinical difference in the decision made by individuals at these extremes. However, the model that is accurate in predicting probabilities between the 0.2 - 0.8 range will be more useful in a clinical setting compared to the model that predicts probabilities well at the extremes. Hence, AUC may be a poor measure of performance for risk prediction models in certain clinical scenarios (Additional file 1: Figure S1). Importantly, the increase in AUC value may not be significant even when the new predictor is statistically associated with the response [9]. To alleviate the problem of low power, the net reclassification index [10] was proposed. However, several concerns have been raised regarding its appropriate use, interpretation, and associated high false positive rates [11, 12], suggesting the need for alternate measures for model comparison. One of the suggested methods was decision curve analysis (DCA) [13, 14].

DCA is used to evaluate the performance of prediction models in clinical decision making. The typical scenario for the application of DCA is when patients have symptoms that suggest a disease but they have not yet been diagnosed with the disease. The clinician has to make a decision regarding whether a biopsy/screening should be performed to diagnose the disease. The biopsy or other screening procedure is associated with various risks or side effects. The decision thus depends on the probability of the disease for that patient, the patient’s preferences, the possible side effects and the clinician’s experience. If the probability of the disease is too high or too low, the decision is generally clear. DCA provides a way to assess the performance of a model in a specific range of interest. DCA has been extensively used to compare competing methods in several diseases [15–17].

DCA is based on the computation of the net benefit for a model. The decision curve computed using the net benefit details the performance of the model at a given threshold probability or in a range of threshold probabilities that is of interest to the clinician making the decision. In several situations, when the net benefit curves for 2 competing models cross in the range of interest, it is difficult to select the best model. There is no available summary measure that can determine the better model. The key deterrent for using the area under the net benefit curve as the summary measure is the assumption that the threshold probabilities would need to follow a uniform distribution. And, if they do not follow a uniform distribution, additional data such as the exact threshold probabilities and patient preferences need to be collected to estimate the threshold probabilities, which limits the application of DCA to data sets that lack these additional data [18].

In this manuscript, we propose a novel way to estimate the distribution of threshold probabilities without collecting additional data by using only a binary clinical decision made by the clinician (e.g., whether screening is performed or not based on the disease probability) that is readily available for most of the data sets. Using the estimated distribution of threshold probabilities, we propose the weighted area under the net benefit curve in the range of interest as a summary statistic for model comparison. We performed several simulation studies to demonstrate the improvement in model comparison for the weighted area under the net benefit curve statistic compared to the standard method that uses confidence intervals to assess whether one model is statistically better than another [13].

## Methods

### Clinical scenario for decision curve analysis

Our guiding example will be the same clinical scenario used by Vickers and Elkin for DCA [14]. Individuals with prostate cancer face the possibility that the cancer could invade either one or both of their seminal vesicles, a condition described as seminal vesicle invasion (SVI). However, SVI is not officially diagnosed until after surgery, following an examination of the surgical sample by a pathologist. Hence, the surgeon has to make a decision regarding the removal of seminal vesicles before prostate surgery, based on the predicted probability of SVI. Several models have been proposed for assessing the probability of SVI prior to prostate surgery, based on predictors such as prostate specific antigen (PSA) and Gleason score (GS) [19, 20]. After estimating the probability of SVI using one of the risk prediction models, the clinician or the patient has a decision to make regarding whether or not the seminal vesicles will be removed during surgery. If the probability of SVI is low, the tip of the seminal vesicles is preserved in surgery to prevent long-term loss of urinary continence [21]. If the probability of SVI is high, and the seminal vesicles are not removed, there is a risk of recurrence of prostate cancer. The decision to remove the seminal vesicles is made using a threshold probability *p*_*t*_, which depends on many factors such as the preference of the patient and the clinician and other covariates such as the age of the patient. If the predicted probability of SVI is greater than the threshold probability, *p*_*t*_, then the seminal vesicles are removed.

### Data simulation

The data for the simulation were based on the clinical scenario for predicting SVI in prostate cancer. We considered a cohort of *n* patients with prostate cancer. For simulation purposes, we assumed the risk of SVI depends on GS [22, 23], PSA [22, 23], and a generic covariate labeled X_1_ (additional covariates such as age, body mass index, and ethnicity can be added to this model). We simulated PSA using an exponential distribution because, typically, PSA levels in prostate cancer patients are heavily skewed towards larger values, which can be simulated using a heavy tailed distribution. We chose a rate parameter of 0.1 to correspond closely to prostate cancer cohorts [24]. We simulated primary and secondary grades for tumors using binomial distributions. The final GS values in the range of 2 to10 were obtained by adding the primary and secondary grades (GS = 2+ binomial (*n* = 4, *p* = 0.5) + binomial (*n* = 4, *p* = 0.5)). The mean for the GS score was 6, which corresponds closely to the average GS score for prostate cancer cohorts [25]. Finally, we simulated *X*_1_ using a normal distribution, with a mean of 27 and a standard deviation of 6. We modeled *X*_1_ based on the values for the mean and standard deviation of the BMI from the 2010 US census. Let *Y* = 1 and *Y* = 0 correspond to the presence and absence of SVI, respectively, in the cohort of *n* prostate cancer patients, and let *p*_*d*_ denote the probability of SVI (*p*_*d*_ = *P* [*Y* = 1]). The simulation model is as follows:

1 $$ logit\left({p}_d\right)=-10+0.1PSA+0.2{X}_1+0.5GS $$

We calculated the probability of SVI in individuals with prostate cancer using this model. We needed to simulate another decision indicator *Z* = [0,1], which indicates whether the clinician or the patient decided to have the seminal vesicles removed. This is dependent on the threshold probability *p*_*t*_. The distribution of *p*_*t*_ in the population is generally unknown. However, *p*_*t*_ is likely to be on the lower side for diseases with serious consequences and to be higher for diseases with minimal consequences. For simulation purposes, we simulated *p*_*t*_ using a beta distribution, *Beta* (2,7). If the disease probability *p*_*d*_ was greater than or equal to the threshold probability *p*_*t*_, the decision would be made to remove the seminal vesicles during surgery (*Z* = 1), otherwise the seminal vesicles would not be removed (*Z* = 0). The above simulation process was used for all the data simulations we report here, with changes to the distribution of *p*_*t*_ and the addition of new predictors for SVI based on the simulation scenario. The number of needed replicates was determined by using a method proposed in [26]. Using the desired precision (half width of the 95 % confidence interval) to be 5, we needed 743 replicates. Therefore, all simulation results are based on 1000 replicates of a cohort of 10000 individuals.

### Data analysis

The purpose of DCA in this study was to compare 2 competing models for the prediction of SVI. We assume 2 models M1 and M2 for predicting the probability of SVI. We used the net benefit to compute decision curves for the 2 competing models. The net benefit [27] was defined as

$$ Net\; Benefit=\frac{True\; Positives}{n}-\frac{False\; Positives}{n}\left(\frac{p_t}{1-{p}_t}\right), $$

where, *p*_*t*_ is the threshold probability and *n* is the total number of individuals.

The general approach for DCA involves computing the net benefit for the 2 models and selecting the model that has higher net benefit at a particular threshold *p*_*t*_ or in a range of thresholds [14]. The standard method uses confidence intervals for the net benefit curve to assess whether one model is statistically better than another [13]. We implemented this approach as described below. To evaluate the confidence intervals, we first resampled the data set with replacement *K* times. We then used these *K* data sets to estimate *K* corresponding net benefit curves. The confidence interval for the net benefit curves at each probability threshold *p*_*t*_ was estimated using α/2 and 1−α/2 percentiles for the bootstrap distribution for a confidence interval coverage of 1−α.

When comparing 2 models using DCA, it is recommended to use the same bootstrap samples for calculating the net benefit for the 2 competing models in order to produce accurate and shorter confidence intervals [13]. Hence, the difference in the net benefit curves for the 2 models as a function of *p*_*t*_ is the statistic used for model comparison. Two models are said to be equivalent if the confidence interval for the difference in the net benefit curves includes zero, we refer to this standard approach as C-NBC. This decision can be made at a particular threshold probability or in a range of threshold probabilities of interest. However, in some situations (i.e., if model 1 is better for some values of *p*_*t*_ and model 2 is better for other values of *p*_*t*_ in the range of interest), it is difficult to identify the best model. Therefore, we initially propose the area under the net benefit curve (A-NBC) as a summary statistic for the performance of the model in the range of threshold probabilities of interest. When comparing 2 models using A-NBC, the statistic of interest is the difference in the area under the net benefit curves for the 2 competing models. The area under the net benefit curve in a range of *p*_*t*_ is computed using trapezoidal numerical integration. Two models are said to be equivalent if the difference in the A-NBC includes zero. The confidence intervals for the A-NBC statistic were obtained from the bootstrap distribution of the statistic.

### Drawback to using the area under the net benefit curve

The drawback to using the A-NBC as a summary statistic is that the integral used to calculate the area assumes that the threshold probabilities are uniformly distributed in the range of interest [18]. In most of the clinical decision making scenarios, this is not a reasonable assumption. For example, most individuals would have lower values of *p*_*t*_ for highly malignant diseases (e.g., cancer) and higher values of *p*_*t*_ for comparatively harmless diseases (e.g., appendicitis). Hence, the distribution of *p*_*t*_ will depend on the disease, cost and benefits of the treatment and patient characteristics such as age, sex, etc. Because the A-NBC statistic does not utilize this information, a clinical decision using the A-NBC may not be practically optimal. By attributing the same weight to model performance at all threshold probabilities, the A-NBC statistic over weights the performance of the model when the clinical significance is comparatively lower and underweights the performance of the model at threshold probabilities where the clinical significance is higher. To overcome this obstacle, we proposed a novel method to estimate the distribution of *p*_*t*_ without any additional data, and calculated the weighted area under the net benefit curve based on the distribution of *p*_*t*_ to obtain improved estimates of model performance.

### Estimating the distribution of *p*_*t*_

The individual threshold probabilities are generally not available in existing datasets. However, one can estimate the distribution of *p*_*t*_ using the clinical decision of *Z* (i.e., removing or not removing the seminal vesicles during prostate surgery), and the predicted probability of SVI (*p*_*d*_). The detailed derivation of the cumulative probability distribution of *p*_*t*_ is provided in the Appendix. Briefly, the cumulative probability distribution of *p*_*t*_ can be expressed as

$$ P\left({p}_t\le k\right)=P\left(Z=1,{p}_t\le k\right)+P\left(Z=0,{p}_t\le k\right). $$

The cumulative probability distribution for individuals who choose to have their seminal vesicles removed can be expressed as

$$ P\left(Z=1,{p}_t\le k\right)=P\left(Z=1,{p}_d\le k\right)+\frac{P\left( pt\le k\right)}{P\left( pt\le pd\right)}P\left(Z=1,{p}_d>k\right). $$

Similarly, the cumulative probability distribution for individuals who choose not to have their seminal vesicles removed can be expressed as

$$ P\left(Z=0,{p}_t\le k\right)=P\left(Z=0\right)-P\left(Z=0,{p}_d>k\right)-\frac{1-P\left({p}_t\le k\right)}{1-P\left({p}_t\le {p}_d\right)}P\left(Z=0,{p}_d\le k\right). $$

Using the above equations, the final cumulative distribution of the threshold probability *p*_*t*_ can be expressed as

2 $$ \begin{array}{l}P\left({p}_t\le k\right)=P\left(Z=1,{p}_d\le k\right)+\frac{P\left({p}_t\le k\right)}{P\left({p}_t\le {p}_d\right)}P\left(Z=1,{p}_d>k\right)+P\left(Z=0\right)-P\left(0,{p}_d>k\right)\\ {}\kern4.08em -\frac{1-P\left({p}_t\le k\right)}{1-P\left({p}_t\le {p}_d\right)}P\left(Z=0,{p}_d\le k\right)\end{array} $$

**Fig. 1:**
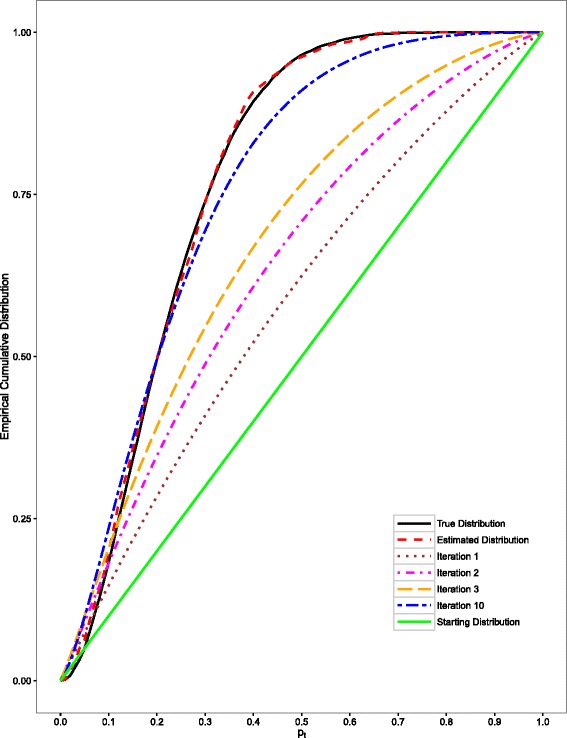
Iterative steps involved in estimating the distribution of threshold probability *p*_*t*_ simulated using a beta distribution. The starting distribution is uniform; the intermediate distributions are shown for iterations 1, 2, 3 and 10; and the final estimated distribution computed after 100 iterations is equivalent to the true distribution

It is complicated to compute the solution to this equation by using traditional methods. Hence, we propose to use an iterative approach to infer the distribution. In our implementation, we used a uniform distribution as the initial estimate of the distribution of *p*_*t*_. After initializing the starting distribution, the distribution of *p*_*t*_ is updated using equation (2). In the next iteration, the estimated distribution of *p*_*t*_ is used as the input value to equation (2). The process is repeated until the distribution converges (see Fig. 1).

### Weighted area under the net benefit curves

After estimating the distribution of *p*_*t*_, we proposed the weighted area under the net benefit curve (WA-NBC), as an improved summary statistic for comparing risk prediction models. The WA-NBC statistic is calculated as

$$ \mathrm{W}\mathrm{A}-\mathrm{N}\mathrm{B}\mathrm{C}={\displaystyle {\int}_{p_t}NBC\left({p}_t\right)}d{p}_t, $$

where *NBC* (*p*_*t*_) is the net benefit curve for the corresponding model, *f* (*p*_*t*_) is the density of *p*_*t*_ and the integration is over the range of interest of threshold probabilities. When 2 competing models are compared using the WA-NBC method, the statistic of interest is the difference in the WA-NBC statistics that correspond to the 2 models. The confidence intervals for the statistic were obtained using the standard bootstrap approach.

## Results

We used 3 different approaches, C-NBC; A-NBC; and WA-NBC to compare 2 competing risk prediction models in the range of interest of threshold probabilities.

### Simulation 1: Type 1 error and power

The type 1 error rates were based on the comparison of models M1 and M2. M1 included predictor variables PSA, GS, and X_1_ as in equation (1) and 3 non-causal predictors (i.e., coefficient zero). M2 included PSA, GS, and X_1_ as in equation (1) and 3 different non-causal predictors. The purpose of including different non-causal predictors in M1 and M2 was to evaluate the type 1 error rate when 2 models were equivalent but not identical. Therefore, we considered 2 models, M1 and M2, which had identical causal variables but different non-causal variables. In this simulation scenario, the models are equivalent, *M*1 ≡ *M*2, because the non-causal predictors have an effect size of zero. All the non-causal predictors were simulated using the standard normal distribution. The type 1 errors and powers were calculated using the bootstrap confidence interval approach. The type 1 errors for the 3 different approaches, C-NBC, A-NBC, and WA-NBC, were well controlled.

To assess power, the probability of SVI was simulated using the model

$$ logit\left(P\left(Y=1\right)\right)=-10+0.1PSA+0.2{X}_1+0.5GS+\gamma {n}_1+\mathit{\in}. $$

The power for the methods was based on comparing the 2 models, M1 and M2, where M1 included all risk factors, PSA, X_1_, GS, and *n*_1_ and 2 non-causal predictors, *n*_2_,*n*_3_. M2 included PSA, X_1_, and GS, but not *n*_1_, and 3 additional independent, non-causal predictors. The predictors *n*_1_,*n*_2_…,*n*_6_ were simulated using a standard normal distribution. M1 was superior to M2 because it included all the causal predictors. To compare the competing models, we simulated data using 3 values of γ (0.3,0.35 and 0.4) to illustrate the change in power while varying the effect of the causal predictor. For this simulation scenario, the range of interest for *p*_*t*_ was considered to be between 30 % to 50 % (i.e., we compared the performance of the methods in this range of *p*_*t*_). The power for the C-NBC method was computed for threshold probabilities within the range of interest and averaged over the range (Table 1).

At γ=0.3, the statistical power achieved when using the C-NBC method was 0.31, which was lower than that achieved by the proposed WA-NBC method (0.53) at the 0.05 level of significance. For γ = (0.35,0.4) the power achieved by using the C-NBC method (0.46, 0.61) was lower than the power for the WA-NBC method (0.68, 0.79).

### Simulation 2: Convergence of the iterative process of estimating the distribution of *p*_*t*_

The patient threshold probabilities were modeled using a beta distribution *Beta* (2,7). The simulated data were used to estimate the distribution of the threshold probabilities using the recursive method (detailed in Methods). At each iteration, the estimated distribution of the threshold probabilities *p*_*t*_ approaches the true distribution of *p*_*t*_. The final estimated distribution of *p*_*t*_ computed after 100 iterations converged to the original simulated distribution of threshold probabilities, as shown in Fig. 1. The convergence to the original simulated distribution of threshold probabilities when the patient threshold probabilities were modeled using a truncated exponential distribution (rate parameter 10 and truncated to the right at 1) is shown in Additional file 1: Figure S2.

### Simulation 3: Impact of weighting the net benefit using the distribution of *p*_*t*_

The following simulation shows the importance of using the estimated distribution of *p*_*t*_ that we proposed to use in calculating the WA-NBC statistic compared to simply using a uniform distribution for *p*_*t*_ that is used to calculate the A-NBC. The statistic of interest is the total net benefit for all the patients in the cohort, which summarizes the performance of a model for the cohort. We compared the total net benefit in 2 scenarios: 1) when the true threshold probabilities (*p*_*t*_) were uniformly distributed; and 2) when the true threshold probabilities (*p*_*t*_) were distributed as a beta distribution (Beta (2,7)). The model

$$ logit\left(P\left(Y=1\right)\right)=\beta {}_0+{\beta}_1PSA+{\beta}_2{X}_1+{\beta}_3GS $$

was fitted to the training data set and the coefficients were estimated. The test data were used to calculate the net benefit curve for the model. The total net benefit for the cohort was assessed for the test data set using 3 methods: 1) Using the true values of *p*_*t*_ used in the simulation; 2) using the estimated distribution of *p*_*t*_; and 3) using a uniform distribution. We simulated 1000 replicates to evaluate the confidence intervals for the total net benefit using the 3 methods (Table 2).

When the threshold probabilities were simulated using a uniform distribution, the net benefit obtained by using the estimated distribution compared to the uniform distribution was 1685.7 (1682.0–1689.3) compared to 1689.5 (1686.4–1692.5), respectively, which, as expected, was equivalent to the true net benefit of 1689.4 (1686.5–1692.3) obtained using the true values of *p*_*t*_ in the simulation. However, when the threshold probabilities were simulated using a beta distribution, the net benefit obtained by using the estimated distribution compared to the uniform distribution was 3013.9 (3010.5–3017.2) compared to 1692.1 (1689.2–1695.0), respectively. In this scenario, the true net benefit for the cohort, obtained using the true values of *p*_*t*_ used in the simulation, was 3013.7 (3010.3–3017.2), which is closer to the net benefit computed using the estimated distribution of *p*_*t*_. The net benefit computed using the uniform distribution underestimated the total net benefit of the method and therefore underestimated model performance.

### Simulation 4: Example showcasing the utility of WA-NBC

The threshold probabilities were simulated from a beta distribution (Beta (2,7)). In this analysis, the performance of the 2 models M1 and M2 was compared in the *p*_*t*_ range of 15 % to 45 %. The models are

$$ \begin{array}{l}\mathrm{M}\mathrm{odel}\ \mathrm{M}1: logit\left(P\left(Y=1\right)\right)={\beta}_0+{\beta}_1PSA\\ {}\mathrm{M}\mathrm{odel}\ \mathrm{M}2: logit\left(P\left(Y=1\right)\right)=I\left(GS\ge 6\; and\;{X}_1>25\right).\end{array} $$

**Fig. 2:**
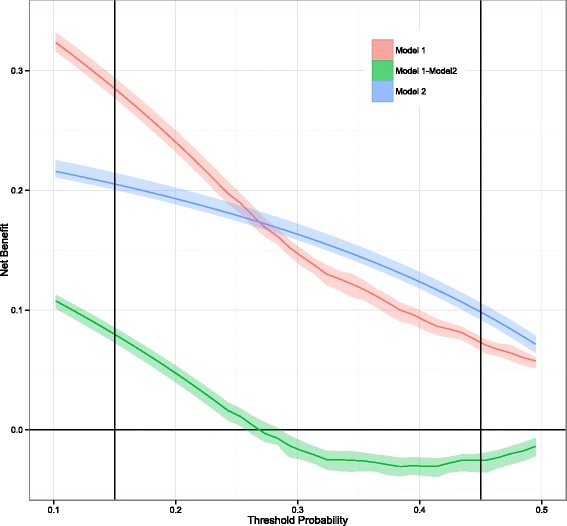
Net benefit curves for models M1 and M2, along with their confidence intervals. The range of interest is from 0.15–0.45 as indicated by the *vertical lines*. This example showcases that model 1 is better than model 2 for study participants even though model 2 is better than model 1 for most of the risk thresholds (0.27–0.45) in the range of interest

Net benefit curves were constructed for each of the models, along with confidence intervals (Fig. 2). As the 2 models cross in the region of interest, we cannot determine whether M1 > M2, M2 > M1, or M1 ≡ M2. We used the WA-NBC method and A-NBC method to evaluate the model performances in the range of interest. Using the A-NBC method, the confidence interval for the test statistic was (−0.0012, 0.0041), which includes zero, thus implying that M1 and M2 are equivalent in the range of interest. However, using the WA-NBC method, the confidence interval for the test statistic was (0.0064, 0.0157) which is above zero, thus implying that M1 is superior to M2 in the range of interest. As the threshold probabilities were simulated form a beta distribution that is right-skewed, most of the individuals would have lower threshold probabilities. And because model M1 is superior to model M2 at lower threshold probabilities (Fig. 2), most individuals would benefit from using model M1 compared to model M2, which is reflected in our analysis using WA-NBC. Thus, our simulation demonstrates the utility of the weighted area under the curve statistic in a particular situation when one cannot determine the best model using traditional DCA.

## Discussion

In this paper, we present a novel method for estimating the distribution of threshold probabilities for individuals in clinical scenarios. This work was motivated by the absence of a straightforward way to compare 2 risk prediction models when the decision curves cross in the range of threshold probabilities of interest. The key deterrent for the use of a simple summary measure such as the area under the net benefit curve is its unrealistic assumption that the threshold probabilities are uniformly distributed in the range of interest. That assumption is largely unrealistic as the threshold probabilities depend on the given disease and other patient characteristics. For malignant diseases, these probabilities are right-skewed and for non-life-threatening diseases, they are left-skewed.

It is important to note that the individual values of *p*_*t*_ cannot be estimated without additional data; however, the distribution of the values of *p*_*t*_ can be estimated using existing data by solving the recursive equation for cumulative distribution of *p*_*t*_. The recursive equation does not have a closed form solution because the cumulative distribution function is present in both the numerator and denominator in different forms. Therefore, an iterative solution was adopted to solve the equation to estimate the cumulative distribution of *p*_*t*_. The rate of convergence was quick for several forms of hypothesized distributions of *p*_*t*_ (i.e., beta, uniform and truncated exponential) and almost always converged within 100 iterations, which took less than 3 min on a computer with a single processor and a speed of 3.4 GHz. We then used the estimated distribution to propose the weighted area under the net benefit curve as a novel summary measure for model comparison in the range of threshold probabilities of interest.

We performed several simulations to assess the performance of the proposed WA-NBC statistic and compared it to A-NBC and the standard approach, C-NBC. The type 1 errors were well controlled for all the methods. The statistical power for WA-NBC was higher than the power achieved when using the C-NBC method. We also showed that the total net benefit for the cohort obtained by using the estimated distribution was closer to the true total net benefit compared to using the uniform distribution to calculate the area under the net benefit curve. Thus, using the estimated threshold probability distribution accurately quantifies the model performance.

To demonstrate the utility of the weighted area under the curve, we simulated a scenario in which 2 competing models, M1 and M2, cross in the region of interest. In this scenario, it was difficult to make a decision using the C-NBC method. Using the A-NBC method led to the false conclusion that M1 was equivalent to M2, and using the proposed WA-NBC method provided the correct conclusion that M1 was superior to M2. The proposed weighted area under the net benefit curve is a superior measure for comparing risk prediction models when there is a crossover of net benefit curves in the region of interest. Importantly, we recommend using this measure even when the curves do not cross because this measure weights the net benefit curve with respect to the distribution of *p*_*t*_, leading to a more practical estimation of the net benefit for future study participants.

Traditional measures of prediction such as AUC and NRI have limited value for risk prediction in clinical scenarios as they do not account for the cost of the treatment and the associated side effects. The proposed methodology based on the net benefit curves can be easily extended to include the cost and side effects by using a novel risk prediction model with additional predictors. The net harm corresponding to the additional predictors or existing predictors can be incorporated into the net benefit curve estimation using the following definition of net benefit [27]:

$$ Net\; Benefit=\frac{True\; Positives}{n}-\frac{False\; Positives}{n}\left(\frac{p_t}{1-{p}_t}\right)-Net\; Harm $$

The net harm can also include cost effectiveness of the risk prediction models and other measures of utility. Accounting for the net harm will provide a more practical measure to identify the model that is most relevant to a particular clinical scenario.

The proposed methodology is generally used to compare 2 predefined risk prediction models. However, we can improve the efficiency of decision curve analysis using relative utility curves [28]. Relative utility curves are based on the contribution of the risk prediction model to clinical utility compared to a hypothesized perfect prediction. The test trade off, which is analogous to the net harm (discussed above) for net benefit curves, can then be used to evaluate the practical utility of the models when using relative utility curves. The main contribution of the proposed methodology is to recursively estimate the distribution of *p*_*t*_, and subsequently use a weighted summary measure. This framework can be used to improve the performance of relative utility curves in the same manner as net benefit curves.

## Conclusions

We have proposed a novel way to estimate the distribution of threshold probabilities without any additional data. Using the estimated distribution of threshold probabilities, we proposed the weighted area under the net benefit curve as a novel summary statistic for model comparison. We performed several simulation studies to demonstrate the improvement in model comparison using the weighted area under the net benefit curve statistic compared to the standard net benefit curves in various scenarios.

## Abbreviations

A-NBC, Area based net benefit curve method; AUC, Area under the curve; C-NBC, Confidence interval based net benefit curve method; DCA, Decision curve analysis; PSA, Prostate specific antigen; GS, Gleason score; SVI, Seminal vesicular invasion; WA-NBC: Weighted area based net benefit curve method

## Additional file

Additional file 1: Supplementary Material.docx. Includes the Appendix and two supplementary figures referred in the manuscript. (DOCX 327 kb)
