# Supplementary material for: Using the weighted area under the net benefit curve for decision curve analysis
Source: BMC Med Inform Decis Mak. 2016 Jul 18;16:94. doi: 10.1186/s12911-016-0336-x (PMC4949771; doi:10.1186/s12911-016-0336-x)
Supplement: Supplementary file 1 — Supplementary Material.docx. Includes the Appendix and two supplementary figures referred in the manuscript. (DOCX 327 kb) [file 12911_2016_336_MOESM1_ESM.docx]

**Supplementary Material**

**Appendix 1: Derivation of the cumulative distribution of** $\boldsymbol{p}_{\boldsymbol{t}}$

The cumulative distribution of $p_{t}$ can be estimated using the predicted probability of SVI ($p_{d}$) and the clinical decision *Z,* which implies whether or not $p_{t}\leq p_{d}$.

The cumulative probability distribution of $p_{t}$ can be expressed as

$$P\left( p_{t}\leq k \right)=P\left( Z=1,p_{t}\leq k \right)+P\left( Z=0,p_{t}\leq k \right). (A1)$$

The first term in equation (A1) can be expressed as

$$P\left( Z=1,p_{t}\leq k \right)=P\left( Z=1,p_{t}\leq k,p_{d}\leq k \right)+P\left( Z=1,p_{t}\leq k,p_{d}>k \right). (A2)$$

We write the first term in equation (A2) as

$P\left( Z=1,p_{t}\leq k,p_{d}\leq k \right)=P\left( Z=1,p_{d}\leq k \right)$

because $Z=1$ implies $p_{t}\leq p_{d,}$ which in turn implies $p_{t}\leq k$.

The second term in equation (A2) can be expressed as

$P\left( Z=1,p_{t}\leq k,p_{d}>k \right)=P(p_{t}\leq k|Z=1,p_{d}>k)P(Z=1,p_{d}>k)$.

The probability $P(p_{t}\leq k|Z=1,p_{d}>k)$ is the probability of $p_{t}\leq k$ divided by the total probability of $p_{t}\leq p_{d}$. Therefore,

$P\left( p_{t}\leq k | Z=1,p_{d}>k \right)=\frac{P\left( p_{t}\leq k \right)}{P\left( p_{t}\leq p_{d} \right)}$.

Thus, the first term in equation (A1) can be expressed as

$P\left( Z=1,p_{t}\leq k \right)=P\left( Z=1,p_{d}\leq k \right)+\frac{P\left( p_{t}\leq k \right)}{P\left( p_{t}\leq p_{d} \right)}P(Z=1,p_{d}>k)$.

The quantity $P\left( Z=1,p_{t}\leq k \right)$ is estimated as the observed number of individuals for whom the predicted probability of SVI ($p_{d}$) was less than or equal to k among those who chose to have their seminal vesicles removed, divided by the total number of individuals in the study. We similarly estimated the quantity $P(Z=1,p_{d}>k)$.

Similarly, the second term in equation (A1) can be expressed as

$P\left( Z=0,p_{t}\leq k \right)=P\left( Z=0 \right)-P\left( Z=0,p_{d}>k \right)-\frac{1-P\left( p_{t}\leq k \right)}{1-P\left( p_{t}\leq p_{d} \right)}P(Z=0,p_{d}\leq k)$*.*

Finally, the cumulative distribution of the threshold probability $p_{t}$ can be recursively expressed as

$$P\left( p_{t}\leq k \right)=P\left( Z=1,p_{d}\leq k \right)+\frac{P\left( p_{t}\leq k \right)}{P\left( p_{t}\leq p_{d} \right)}P\left( Z=1,p_{d}>k \right)+P\left( Z=0 \right)-P\left( Z=0,p_{d}>k \right)-\frac{1-P\left( p_{t}\leq k \right)}{1-P\left( p_{t}\leq p_{d} \right)}P\left( Z=0,p_{d}\leq k \right) .$$

The recursive equation does not have a closed from solution because the cumulative distribution function is present in both the numerator and denominator in different forms. Therefore, the equation is mathematically intractable and an iterative procedure was used to solve the equation for the cumulative distribution of $p_{t}$. The cumulative distribution function at the (i+1)^th^ iteration is computed as:

$$P\left( p_{t}\leq k \right)^{\left( i+1 \right)}=P\left( Z=1,p_{d}\leq k \right)+\frac{P\left( p_{t}\leq k \right)^{\left( i \right)}}{P\left( p_{t}\leq p_{d} \right)^{\left( i \right)}}P\left( Z=1,p_{d}>k \right)+P\left( Z=0 \right)-P\left( Z=0,p_{d}>k \right)-\frac{1-P\left( p_{t}\leq k \right)^{\left( i \right)}}{1-P\left( p_{t}\leq p_{d} \right)^{\left( i \right)}}P\left( Z=0,p_{d}\leq k \right) .$$

where, $P\left( p_{t}\leq k \right)^{\left( i \right)}$represents the value of cumulative distribution function at the i^th^ iteration. The iterative procedure is initialized with a uniform cumulative distribution. For computing the cumulative distribution of $p_{t}$, we varied k between 0 and 1, with equally spaced increments of 0.01.


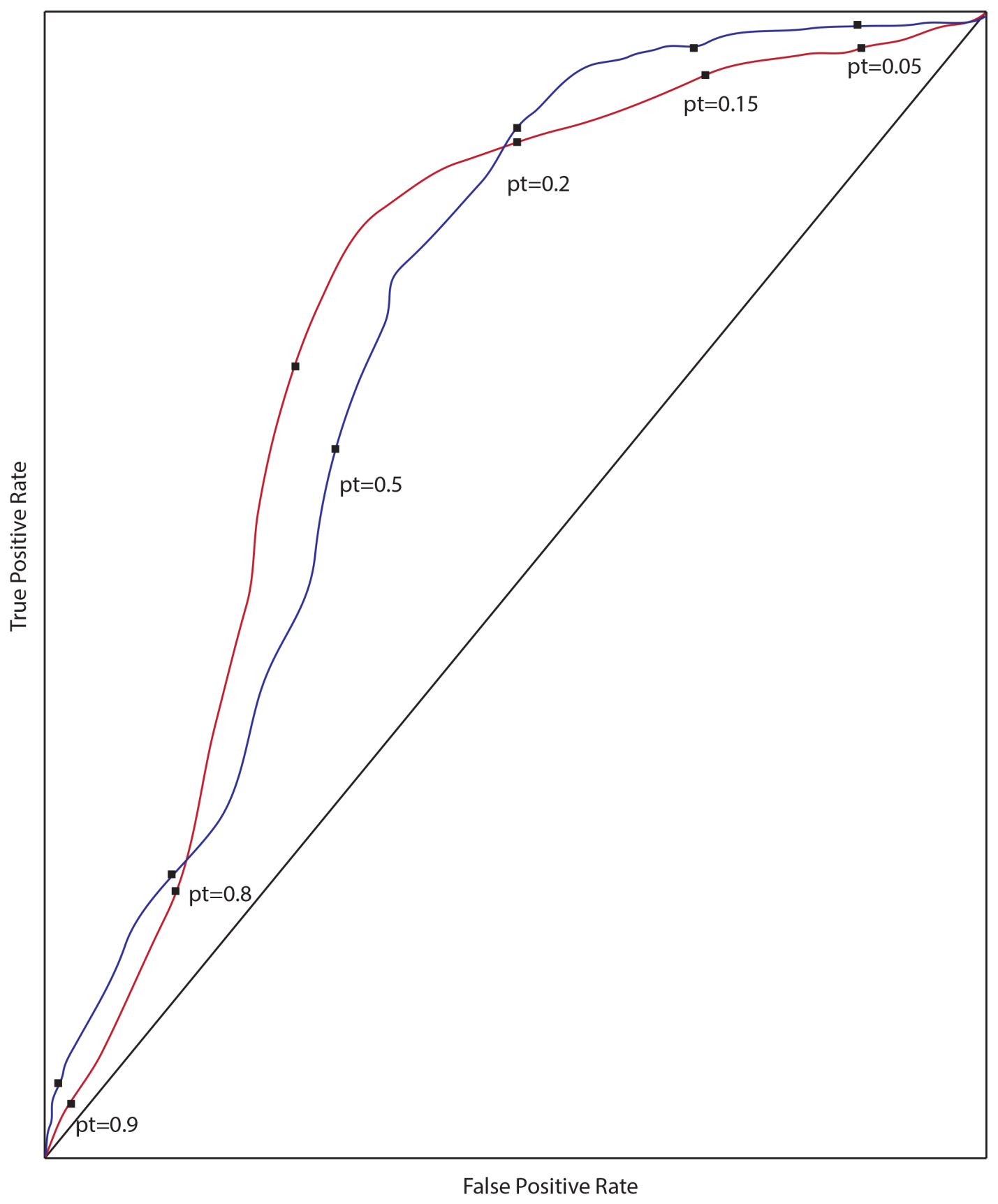


Supplementary Figure S1: This figure depicts the scenario mentioned in the Introduction, where AUC may be a poor measure of performance for risk prediction models in certain clinical scenarios.

**
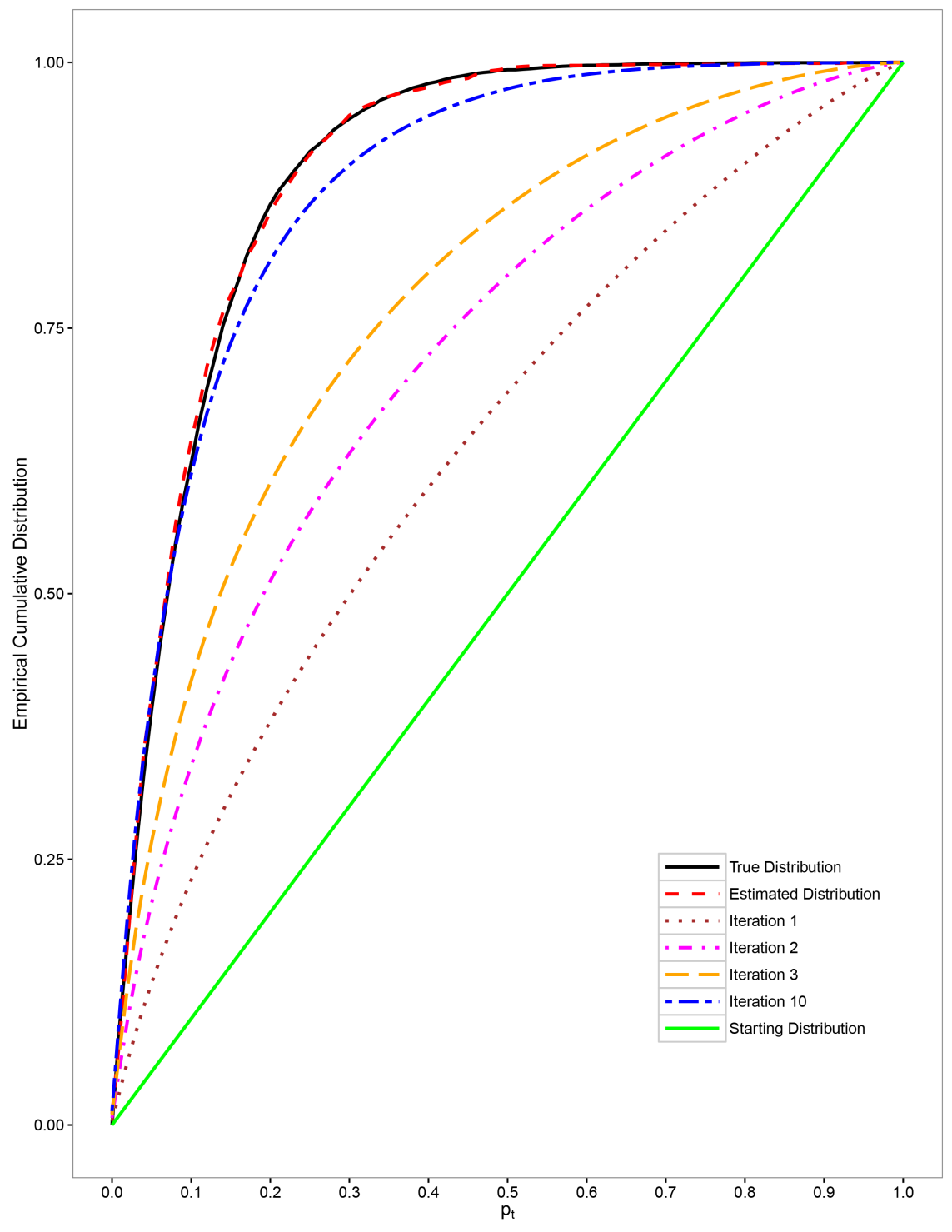
**

Supplementary Figure S2: Iterative steps involved in estimating the distribution of threshold probability $p_{t}$ simulated using a truncated exponential distribution (rate parameter 10 and truncated to the right at 1). The starting distribution is uniform; the intermediate distributions are shown for iterations 1, 2, 3 and 10; and the final estimated distribution computed after 100 iterations is equivalent to the true distribution.
